# Acute Pancreatitis in Pregnancy and Puerperium: Assessing Maternal and Fetal Impact, Etiologies, and Clinical Outcomes at a Tertiary Care Hospital in Pakistan

**DOI:** 10.7759/cureus.76393

**Published:** 2024-12-25

**Authors:** Faryal Anees, Rozilla S Khan, Sumaira Naz, Zahid H Wadani

**Affiliations:** 1 Obstetrics and Gynaecology, Aga Khan University Hospital, Karachi, PAK

**Keywords:** acute pancreatitis in pregnancy, gallstones, hypertriglyceridemia, preeclampsia, preterm birth

## Abstract

Introduction

The association of acute pancreatitis with adverse obstetric outcomes remains subject to great controversy. Outcomes are affected by the standard of care available, and hence, will be better in developed countries than in underdeveloped countries like Pakistan. Therefore, this study aimed to understand the clinical characteristics and treatment of acute pancreatitis in pregnancy (APIP) and its associated maternal and neonatal outcomes in a tertiary care hospital in Pakistan.

Methodology

A retrospective clinical analysis of APIP cases during pregnancy and the postpartum period was conducted at Aga Khan University Hospital (AKUH) in Karachi, Pakistan, from January 1, 2015, to December 31, 2021. Data were collected from the medical records of women diagnosed with APIP. Pregnant women with chronic and recurrent pancreatitis were excluded. We analyzed baseline demographics, obstetric outcomes, and neonatal outcomes for those who delivered at AKUH. These individuals were monitored in postnatal outpatient clinics to track changes in their initial symptoms. Data were analyzed using IBM SPSS version 25.0.

Results

A total of 32 patients with APIP were included. Among them, 13 (40.6%) were primigravida, and 21 (65.6%) were multigravida. The majority 22 (69%) presented in the third trimester. The most common etiology was gallstones 13 (40.6%), followed by idiopathic causes 12 (37.5%), and hypertriglyceridemia 2 (6.3%). The disease course remained mild in 24 (75%) of patients, moderate in 5 (15.6%), and severe in 3 (9.4%) according to The Atlanta Criteria. Conservative management was successful in 27 (84.4%), while 5 (15.6%) of patients required surgical intervention. Severe disease courses led to organ dysfunction and disseminated intravascular coagulation in 5 (15.6%), and maternal mortality in 3 (9.4%). Preeclampsia was the most common obstetric complication (6 (18.8%)), and 13 (40.6%) patient underwent an emergency cesarean section. In the mild group, one patient experienced miscarriage, and 14 (43.7%) patients preterm births, with 6 (18.8%) of these newborns requiring NICU admissions and 2 (6.3%) experiencing perinatal mortality. In the severe group, 2 (6.3%) had intrauterine fetal demise. Post-delivery, 12 (37.5%) were lost to follow-up. However, among the patients who followed up, 21 (62.5%) experienced an improvement in their general health condition.

Conclusions

Our study underscores the increased risk of preeclampsia and preterm births in women with APIP, emphasizing the need for heightened vigilance in disease progression and antenatal care.

## Introduction

Pancreatic inflammation can manifest as either acute or chronic, contingent upon the duration of the initial insult [1]. Acute pancreatitis in pregnancy (APIP) occurs at a frequency ranging from 1 in 1000 to 1 in 10,000 deliveries; however, it can give rise to substantial maternal and fetal mortality and morbidity [2,3]. Recent studies have highlighted an increasing incidence of this serious condition during pregnancy over the past decade, while instances of chronic pancreatitis are rare within the pregnant population [1]. The characteristics of APIP might exhibit variations among diverse geographical regions and ethnic groups due to distinct lifestyles and the availability of healthcare resources [4].

Various factors have been identified as potential causes of acute pancreatitis, with gallstones emerging as the most prevalent factor [1]. Other factors encompass idiopathic origins, hypertriglyceridemia, alcohol consumption, trauma, and certain medications [2,5-7]. Moreover, advanced maternal age, increased parity, elevated body mass index, a high-fat diet, and acute fatty liver of pregnancy have all been proposed as pregnancy-related risk factors for acute pancreatitis [8]. Diagnosing and treating APIP is typically challenging due to the overlap in symptoms between a normal pregnancy and a pregnancy complicated by acute pancreatitis [1-3]. The commonly reported symptoms of APIP include abdominal pain, vomiting, fever, and jaundice [7].

An ongoing debate surrounds the clinical trajectory of APIP and its impact on maternal and neonatal outcomes. In the past decade, the reported rates of maternal and fetal mortality attributed to APIP were notably elevated, often attributed to delays in accurate diagnosis and timely intervention [7]. Several studies propose that APIP might pose a greater threat to the fetus compared to the mother [9,10]. However, the literature also highlights the potential for APIP to complicate ongoing pregnancies, leading to complications like pancreatic necrosis, abscess formation, disseminated intravascular coagulation, venous thromboembolism, multi-organ dysfunction, and critical illness [8]. Importantly, severe acute pancreatitis frequently arises during the third trimester of pregnancy and can be associated with preeclampsia, HELLP (hemolysis, elevated liver enzymes, and low platelets) syndrome, and gestational diabetes. The widespread inflammation and abnormalities at the microvascular level predispose women to preeclampsia and APIP, and which leads to one another is still a debate. These complications elevate the risk of fetal mortality or preterm delivery [2,6,7].

The link between acute pancreatitis and adverse obstetric outcomes is still a debatable subject. The extent to which pregnancy influences the progression of APIP and whether morbidity and mortality are heightened in pregnant women remains uncertain. Existing literature suggests that the outcome is influenced by the quality of accessible healthcare, with developed countries exhibiting better results compared to low- and middle-income nations like Pakistan and India [11]. The focus of this study was to determine the clinical traits, management, and impact of APIP on maternal and neonatal outcomes within our healthcare context.

## Materials and methods

This retrospective study was conducted in the Obstetrics & Gynecology department at the Aga Khan University Hospital, Karachi, Pakistan. After obtaining approval from the Ethical Review Board (ERC # 2022-7123-20358), data was retrieved from the medical records of the women diagnosed with acute pancreatitis during all three trimesters from January 1st, 2015 to December 31st, 2021. The antenatal care and disease course of these patients was analysed. The inclusion criteria included all pregnant women with acute pancreatitis diagnosed during pregnancy and puerperium (6 weeks postpartum). Patients were excluded if had a diagnosis of chronic or a recurrence of pancreatitis in the same pregnancy.

A total of 32 patients met the inclusion criteria. A self-structured proforma was used to collect data from the patients’ medical records and patient care inquiry portal. Information regarding demographic variables, including age, BMI (body mass index), parity, gestational age at the time of presentation, pregnancy course, radiological information, and laboratory workup for acute pancreatitis, was gathered. Details of the severity and clinical course of disease, and the maternal, obstetric, and perinatal outcomes were collected. In patients whose pregnancies resulted in delivery, gestational age at delivery, birth weight, Apgar score, NICU admission, and neonatal outcome were also noted. The medical records were further reviewed for patients who presented in outpatient clinics to assess for improvement or worsening of their symptoms. All personal information of the patients was de-identified.

We used the revised Atlanta classification (RAC) system 2012 to classify the disease into three categories, namely mild, moderately severe, and severe acute pancreatitis, based on the presence and duration of organ failure. This classification standardized the practice by providing diagnostic criteria for the variable presentation of this disease, with the added benefit of uniform grading of the disease severity and classifying local complications. Mild acute pancreatitis has no organ failure and has a self-limiting disease course. Moderately severe disease has transient (less than 48 hours) organ failure with or without local complications. On the other hand, severe acute pancreatitis is defined by persistent and greater than 48 hours disease course along with local and systemic complications [12].

Statistical analysis

All data analyses were performed using IBM Corp. (released 2017. IBM SPSS Statistics for Windows, Version 25.0. Armonk, NY: IBM Corp). Categorical variables were presented as frequencies and percentages and between-group differences were assessed using the Chi-square test or the Fisher exact test, as appropriate. Normally distributed continuous variables were presented as mean± standard deviation (SD) and between-group differences were assessed using one-way analysis of variance (ANOVA) or t-test. Non-normally distributed continuous variables were analyzed using a non-parametric test. All tests were two-tailed, and p-value <0.05 was considered statistically significant.

## Results

During the study period, a total of 37,576 pregnant patients received care at our hospital. Among these, 32 patients were identified with APIP, resulting in an incidence rate of 0.08% within our study population. The average age of the women was 27±4.59 years when APIP initially manifested. Regrettably, 11 of these women were unable to have their weight recorded upon hospital arrival, resulting in the average BMI being calculated from the remaining data, yielding 26 kg/m² with a standard deviation of 4.97 kg/m². Most women (82.8%) experienced symptoms during the third trimester, with an average gestational age of 28.5±7.45 weeks. Notably, no instances of chronic hypertension, diabetes mellitus, or fatty liver disease were identified within our specific population of interest (Table 1).

**Table 1:** Demographic characteristics of pregnant women diagnosed with acute pancreatitis in pregnancy

Variables	n	Estimates
Age (Years)	32	27.1±4.59
BMI (kg/m²)	21	26.0±4.97
Gestational age (Weeks)	29	28.5±7.45
Post-natal cases (within 1-10days after delivery)	3	9.40%
Parity		
Primigravida	11	40.60%
Multigravida	21	65.60%
Previous miscarriages	7	21.90%
Prior comorbidities		
Thalassemia minor	1	3.10%
Obstetric cholestasis	2	6.30%
Hypothyroidism	1	3.10%
Hepatitis B positive	1	3.10%
Chronic kidney disease	1	3.10%
Carcinoid periampullary tumor	1	3.10%

**Figure 1:**
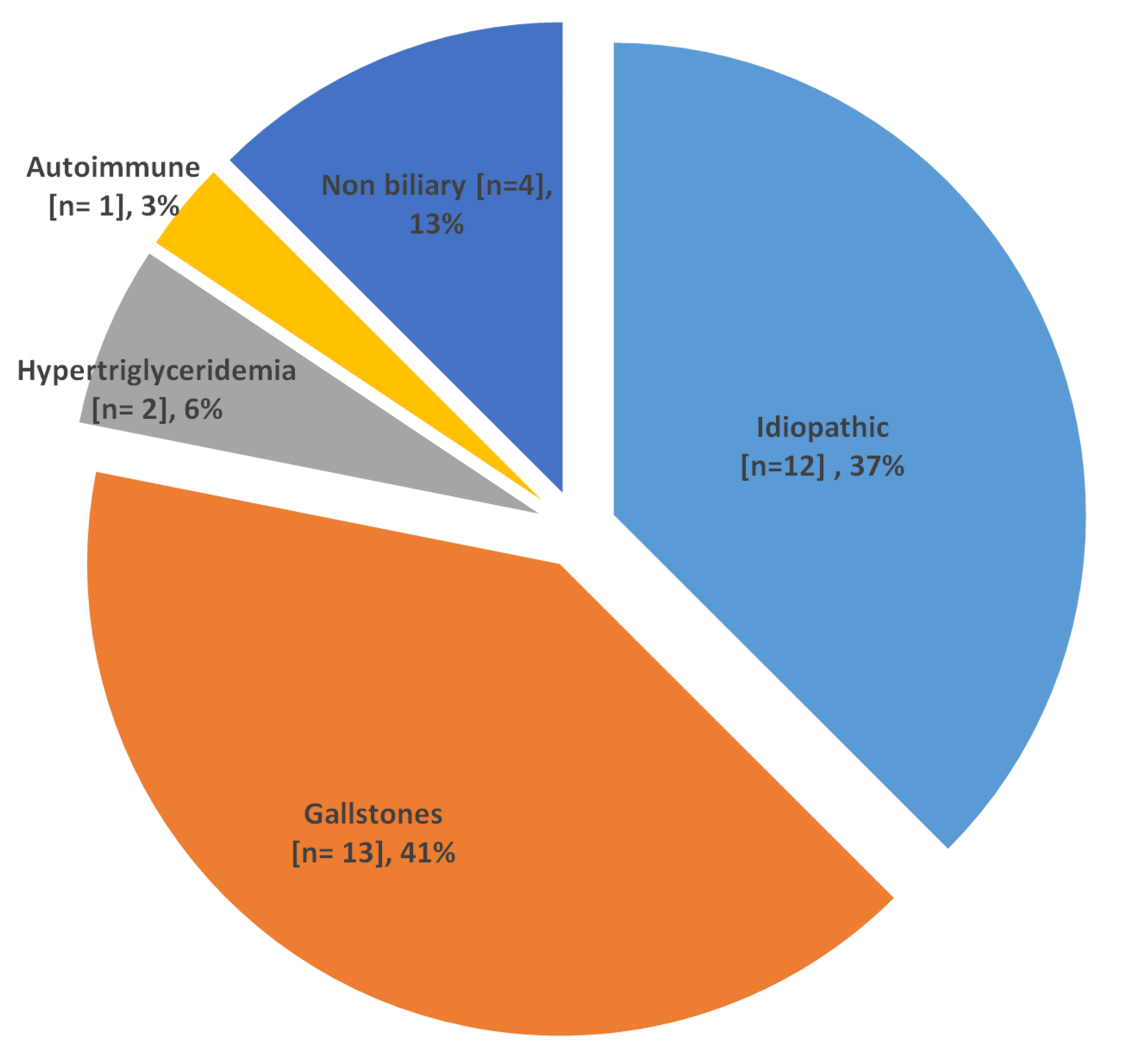
Etiology of acute pancreatitis in pregnancy (total number of patients = 32)

As shown in (Table 2), the primary initial symptom experienced by a majority of the women upon arrival at the hospital was abdominal pain (84.4%), often centered on the epigastrium. A smaller number of individuals mentioned altered bowel habits, including constipation, along with fever. The predominant radiological imaging modality employed was ultrasound (81.3%), followed by CT scan (21.9%) and MRCP (18.8%) in cases where organ dysfunction was observed. Ultrasound examinations revealed findings of gallstones and sludge gallbladder in 11% of cases, while acute pancreatitis was identified in 15%. Notably, 19% of cases displayed normal scan results. Mild cases were managed conservatively, however, few moderate and all severe cases of APIP underwent surgical management. Conservative management with IV fluids, analgesia, and parental nutrition was successful in 84.3% of cases. Among these, 14.8% of patients also underwent intubation due to worsening of their symptoms. Surgical management was given to 15.6% of the women which included laparoscopic cholecystectomy (60%), endoscopic retrograde cholangiopancreatography (ERCP) (20%), and ileal resection with bowel loop anastomosis secondary to ileal perforation with severe pancreatitis (20%). Figure 1 depicts the subsequent leading causes of APIP. Among the patients, the most prevalent cause of the condition was attributed to gallstones, accounting for 41% of cases.

**Table 2:** Clinical symptoms, investigation findings, and treatment offered to the study participants

Variables	n	%
Symptoms at Presentation		
Epigastric pain	27	84.40%
Nausea and vomiting	19	59.40%
Treatment		
Conservative Treatment	27	84.40%
Surgical Intervention	5	15.60%
Investigations		
Ultrasound	26	81.30%
CT scan	7	21.90%
MRCP	6	18.80%

**Figure 2:**
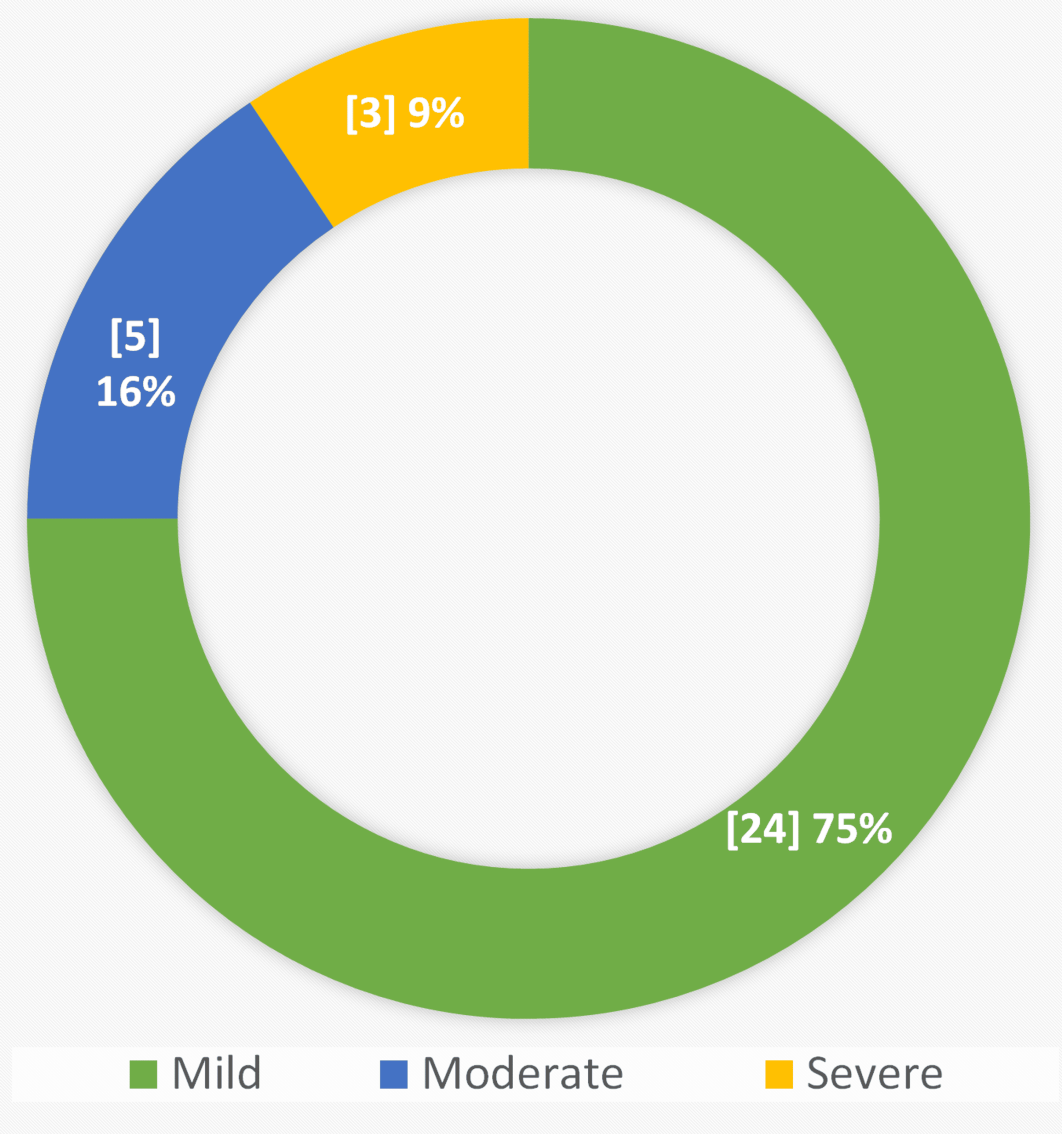
Disease course as per the Revised Atlanta Classification System - 2012 Total number of patients = 32

As shown in Figure 2, the disease course was classified as 75% mild, 15.6% moderate, and 9.4% severe as per the Atlanta criteria which uses early prognostic signs, organ failure, and local complications to define disease severity [13]. The outcomes of the disease course studied as maternal complications and obstetric outcomes, are shown in Table 3. We found that 21.8% of patients had organ dysfunction, followed by acute kidney injury in 71.4%, pulmonary edema in 42.8%, and gastrointestinal bleeding in 14.2% of patients. We found no cases of pancreatic pseudocyst in our study population. The women in whom the pregnancies continued, the obstetric outcomes were studied. Table 3 shows preeclampsia (18.8%) was the most frequently encountered complication. None of our patients developed eclampsia, HELLP syndrome, or had retained placenta. Maternal mortality among the women diagnosed with APIP was calculated up to 9.4%. We found cesarean delivery to be the mode of delivery in the majority of patients (~50%). Unfortunately, 34.3% of the women didn’t deliver in our hospital.

**Table 3:** Maternal complications, obstetrics outcomes, and mode of delivery of study participants

Variables	n	%	
Maternal Complication			
Acute Necrosis / Encapsulated Necrosis	1	3.10%	
Organ Dysfunction	7	21.80%	
Acute Kidney Injury	5	71.4%%	
Pulmonary Edema	3	42.80%	
Gastrointestinal Bleeding	1	14.2%%	
Pleural Effusion	2	6.30%	
Ascites	2	6.30%	
Disseminated Intravascular Coagulation	5	15.60%	
ICU admission	4	12.50%	
Obstetric Outcomes			
Maternal Mortality	3	9.40%	
Pregnancy Induced Hypertension	2	6.30%	
Pre-Eclampsia	6	18.80%	
Gestational Diabetes Mellitus	3	9.40%	
Preterm Pre-labor Rupture of Membranes	1	3.10%	
Placental Abruption	3	9.40%	
Placenta Previa	1	3.10%	
Mode of Delivery			
Spontaneous Vaginal Delivery	3	9.40%	
Elective Cesarean Delivery	3	9.40%	
Emergency Cesarean Delivery	13	40.60%	
Delivered outside	2	6.30%	
Miscarriage	1	3.10%	
Postpartum Hemorrhage	2	6.30%	

We observed 43.7% preterm birth, 15.6% term births and 3.1% miscarriages in women with mild disease. With severe disease, fetal loss of 6.3% was observed. The median birth weight among singleton pregnancies was 2.2kg and 18.8% were shifted to NICU due to prematurity as shown in (Table 4). The perinatal mortality rate was 6.3% among babies of delivered cases.

**Table 4:** Perinatal outcomes among women with acute pancreatitis in pregnancy NICU: neonatal intensive care unit

Perinatal Outcomes	n	%
Miscarriage	1	3.1%
Preterm birth	14	43.7%
Term birth	5	15.6%
Data not available	15	46.9%
Median estimated fetal weight on ultrasound (kg)	21	1.6 kg
Birth Weight (kg)		
Single	14	mean: 2.2 kg ± 0.9
Multiple (triplet)	1	0.67kg ,0.87kg ,0.75kg ( mean : 0.7 ± 0.1)
Weight not reported	13	40%
Intrauterine death	2	6.3%
Stillbirth	0	0.0%
Perinatal mortality	2	6.3%
Apgar Scores at birth		
At 1 min (below 7)	2	6.3%
At 1 min (above or equal to 7)	15	46.9%
At 5 min (below 7)	2	6.3%
At 5 min (above or equal to 7)	15	46.9%
Apgar score not reported	15	46.9%
NICU admission	6	18.8%

Among the delivered patients who followed up for their postnatal visit, 62.5% of the women had improvement in their symptoms whereas 37.5% were lost to follow-up. We compared the laboratory parameters of our study subject’s pre- and post-disease course (Table 5). It was found that serum amylase and lipase were equally raised at the time of diagnosis and improved thereafter with treatment.

**Table 5:** Comparison of laboratory parameters among women with acute pancreatitis in pregnancy during and after pregnancy gm/dl: grams per deciliter; umol/L: micromole per liter; IU/L: international units per liter; mg/L: milligram per liter SGPT=serum glutamate pyruvate transaminase; SGOT=serum glutamic-oxaloacetic transaminase; ALP=alkaline phosphatase; CRP=C-reactive protein

Parameter	n	During Pregnancy	After Pregnancy	t -value	P-value
		Mean ± SD	Mean ± SD		
Hemoglobin (gm/dl)	30	10.73±1.81	9.72±1.72	-2.58	0.01
Hematocrit (%)	30	33.11±5.39	29.94±5.73	-2.56	0.01
Total leucocyte count (cells/µL)	30	14.20±7.73	11.51±5.11	-1.64	0.11
Platelets (10^9^/L)	30	270.97±98.48	301.57±170.49	0.89	0.38
Total bilirubin (umol/L)	20	1.47±2.30	0.81±0.71	-1.39	0.18
SGPT (IU/L)	21	108.29±297.8	104.62±281.83	-0.36	0.72
SGOT (IU/L)	16	185.38±586.85	99.38±232.14	-0.96	0.35
ALP (IU/L)	19	153.16±62.38	148.11±69.11	-0.3	0.76
Amylase (U/L)	18	750.56±920.28	189.39±137.78	-2.82	0.01
Lipase (U/L)	20	762.75±608.36	230±155.32	-4.3	0.001
CRP (mg/L)	6	88.14±97.37	61.55±80.96	-1.65	0.15

## Discussion

Over the years, a paradoxical trend has emerged concerning APIP. This trend involves an increase in the number of diagnosed cases, while simultaneously witnessing a decrease in both perinatal and maternal morbidity and mortality rates associated with this condition. This study aimed to assess the impact of acute pancreatitis on both maternal health and fetal well-being, providing a localized perspective to bridge the gap in regional data. The findings from our study indicated a higher occurrence of acute pancreatitis as pregnancy progresses. Our study categorized clinical outcomes into three distinct groups: maternal, perinatal, and obstetric complications. Within these categories, an elevated risk of preeclampsia and preterm births among women affected by APIP was identified.

Our findings are consistent with previous research, which has shown that the distribution of cases across trimesters follows a distinct pattern. The incidence escalates from 12% during the first and second trimesters to 50% in the third trimester and 38% in the early postpartum phase [2]. Consistent with our results, existing literature underscores cholelithiasis as the primary cause of APIP, accounting for more than 65% of cases [1,13]. Subsequent etiologies include alcohol consumption at 5-10% [3,14], hyperlipidemia at 4-6% [6], hypercalcemia at 7-13% [3], medications such as steroids, azathioprine, and valproic acid [15], and trauma and idiopathic factors at 15% [2,14]. Notably, the prevalence of gallstones in Asian nations, including China, is notably lower than in Western regions [16,17]. In contrast to our study, research in these countries, particularly China, has indicated hypertriglyceridemia as the principal cause of APIP. This variance is attributed to ethnicity, a high-fat diet, and an inclination toward elevated lipid acid levels among South Asian and Eastern women [18].

In the initial presentation, abdominal pain (84.4%) and vomiting (59.4%) were prominent symptoms in these women, mirroring those in non-pregnant individuals. This similarity can be attributed to the higher prevalence of other diseases during pregnancy [7]. Achieving an accurate diagnosis necessitates correlating clinical presentation with biochemical analysis of markers, such as serum amylase and/or lipase, alongside radiological imaging [1,2,7,19]. Research indicates that in comparison to serum amylase, serum lipase boasts heightened sensitivity and a broader diagnostic window [20]. However, our study discovered both markers to be equally elevated in our patients. Ultrasound emerged as the most frequently utilized imaging modality, accounting for 81.3% of cases, compared to other available methods [21].

To assess the severity of the disease, we employed the Atlanta criteria [12], revealing that the majority of cases followed a mild course. Several factors during pregnancy can exacerbate the severity of APIP, influencing maternal and neonatal outcomes. The presence of gallstones, which is the most common underlying factor, and hypertriglyceridemia significantly contribute to inflammation and disease progression. The clinical presentation of APIP varies; mild cases generally involve fewer complications, whereas severe cases are often associated with systemic inflammation and potential organ dysfunction. Obstetric complications, such as pre-eclampsia, markedly increase the risk of adverse maternal and neonatal outcomes. Additionally, maternal characteristics, including multigravidity and pre-existing conditions such as obesity and metabolic disorders, heighten susceptibility to severe disease and complications. Delayed or inadequate management of precipitating factors, such as untreated gallstones or hypertriglyceridemia, can further exacerbate the condition and lead to a more severe clinical course [8,12]. Recognizing these factors is critical for timely diagnosis and targeted interventions to improve outcomes in APIP cases.

Similar to retrospective studies, the management approach for mild APIP was conservative and supportive. Successful treatment encompassed appropriate intravenous fluids, analgesia, and parental nutrition. Nonetheless, instances of severe APIP necessitated hospitalization and intervention through endoscopy or surgery [1,3,6,22]. Initially, surgical intervention was deemed inappropriate for pregnant individuals. However, Stimac et al. and Hernandez et al. proposed that due to recent advancements, laparoscopic cholecystectomy could be considered a viable treatment choice, particularly for pregnant women with recurring pancreatitis or cases where medical management had proven ineffective [3,23].

Sun Lu's study demonstrated notable distinctions between severe APIP and mild APIP groups. Specifically, there were significant variations in terms of intensive care unit (ICU) admission, duration of hospital stay, and local and systemic complications. The inflammatory cascade initiated by pancreatitis triggers widespread cytokine release, resulting in systemic inflammation and subsequent organ dysfunction. Pancreatic inflammation often causes fluid sequestration, leading to hypovolemia, reduced perfusion, and ischemia in critical organs. Reperfusion following ischemic injury may worsen organ damage through oxidative stress. Severe pancreatitis can also induce coagulopathy, causing widespread microthrombi formation that further impairs organ perfusion. Additionally, elevated triglyceride levels may cause lipotoxicity and microvascular complications. Pre-eclampsia compounds these issues by increasing systemic vascular resistance and causing endothelial damage. Within the severe APIP category, these women experienced multiple organ failure, leading to the diagnosis of dysfunction in a minimum of two organs (respiratory failure and renal dysfunction). Consequently, 18% necessitated subsequent ICU admissions similar to our results [24].

Multiple prior studies have conveyed notable maternal and perinatal mortality rates of 20% and 50%, respectively, in relation to APIP [7]. Gangat et al. and Ramin et al. similarly documented corresponding findings in their investigations [25,26]. In contrast, our investigation revealed a decrease in maternal and perinatal mortality rates, reducing to 9.4% and 6.3%, respectively. Our outcomes align with recent research conducted by Stimac et al. and Papadakis et al. [3,27]. This decline can be attributed to enhanced antenatal care, the availability of advanced diagnostic techniques in tertiary care settings, increased awareness, and the ongoing refinement of therapeutic interventions over time. Early diagnosis would enable the healthcare professional to provide early symptomatic care and admission to the hospital where the progression and improvement of the disease can be monitored efficiently and timely action can be taken if worsening of symptoms are noted.

Just like previous studies, the correlation between APIP and conditions like preeclampsia and HELLP syndrome has been observed to complicate ongoing pregnancies, potentially leading to increased feto-maternal mortality or preterm delivery [2, 6,7,28]. Though rates of preeclampsia were high in a subset of our population, we did not observe any cases of HELLP syndrome. Our findings indicated that postpartum hemorrhage (PPH) occurred in 6.3% of cases. An instance highlighted by Liu documented a rare case of APIP complicated by PPH associated with Sheehan syndrome; however, the precise underlying pathophysiology of this association remains uncertain [29]. Xu et al. underscored that APIP can pose a greater threat to the fetus compared to the mother [9].

In line with retrospective investigations, fetal losses in the mild pancreatitis group were attributed to miscarriages and preterm births, while the severe group exhibited instances of fetal death and stillbirth [24]. Our study disclosed rates of 34.4% for preterm births, 6.3% for intrauterine deaths, and 6.3% for perinatal mortalities. Historically, the mode of delivery exhibited varying rates of Cesarean sections, with percentages of 76%, 66%, and 66.7% mostly attributed to fetal distress [30]. Within China, neonatal complications among such patients have also demonstrated an association with neonatal mortality, along with conditions like hyperbilirubinemia, hypoglycemia, respiratory distress syndrome, infectious diseases, and intracranial hemorrhage [10].

As this study was based on retrospective analysis, it has some inherent limitations. Notably, some information was missing. A subset of women received treatment for the disease but did not follow up for the delivery phase. Some cases were lost to follow-up, and despite multiple attempts to reach out to these patients, we were unable to ascertain the ultimate outcomes of their pregnancies, including recurrence or surgery. Similarly, not all patients underwent complete assessments of certain laboratory indices. This study’s findings are constrained by a relatively small sample size, which can be attributed to the lower incidence of APIP within our hospital context.

However, a key strength of our study lies in the fact that the study sample was derived from a substantial dataset of pregnant women and we included data from all the available APIP cases for holistic information. This feature could contribute valuable practical insights to the global understanding of APIP.

## Conclusions

In recent decades, there has been a substantial decline in the incidence of fetal and maternal mortality and morbidity associated with APIP. While diagnosing the condition can be complex, milder cases generally exhibit favorable prognoses. The risk of preeclampsia and preterm delivery is heightened in these individuals. In the context of Pakistan and other lower and middle-income countries, factors such as constrained healthcare resources, unequal medical access, and cultural elements can influence the effects of acute pancreatitis. The majority of the women, in the society that we live in, chose to deliver at home and have no antenatal visits ever in their pregnancies as their culture has taught them that visiting the hospital for antenatal care is unnecessary and is a sign of weakness rather. Therefore, plenty of women present very late to the hospital. 

Pregnancies complicated by APIP require increased vigilance and comprehensive antenatal care to closely monitor the disease progression. Early identification of risk factors is essential in pregnant women presenting with symptoms of pancreatitis, and screening for gallstones, hypertriglyceridemia, and idiopathic causes can reduce complications. This research endeavor has the potential to guide the management of ongoing pregnancies by facilitating timely antenatal care and appropriate clinical interventions that can lead to improved outcomes. Conservative management should be prioritized where possible, but timely surgical interventions are critical for severe cases. Proactive monitoring of obstetric complications is important and healthcare providers should be aware of the increased risk of preeclampsia, emergency cesarean sections, and preterm labor in these patients. A significant proportion of patients did not attend follow-up visits, emphasizing the importance of ensuring continuity of care during the postpartum period. Our findings underscore the need for early diagnosis, close monitoring, and multidisciplinary care in managing APIP during pregnancy to minimize maternal and neonatal morbidity and mortality. Additionally, developing customized approaches that account for the local circumstances becomes crucial for successful public health interventions.
